# Triphala Extract Suppresses Proliferation and Induces Apoptosis in Human Colon Cancer Stem Cells via Suppressing c-Myc/Cyclin D1 and Elevation of Bax/Bcl-2 Ratio

**DOI:** 10.1155/2015/649263

**Published:** 2015-06-17

**Authors:** Ramakrishna Vadde, Sridhar Radhakrishnan, Lavanya Reddivari, Jairam K. P. Vanamala

**Affiliations:** ^1^Department of Food Science, The Pennsylvania State University, University Park, PA 16802, USA; ^2^Department of Biotechnology & Bioinformatics, Yogi Vemana University, Kadapa 516003, India; ^3^Department of Plant Science, The Pennsylvania State University, University Park, PA 16802, USA; ^4^The Pennsylvania State Hershey Cancer Institute, Penn State Milton S. Hershey Medical Center, Hershey, PA 17033, USA

## Abstract

Colon cancer is the second leading cause of cancer related deaths in the USA. Cancer stem cells (CSCs) have the ability to drive continued expansion of the population of malignant cells. Therefore, strategies that target CSCs could be effective against colon cancer and in reducing the risk of relapse and metastasis. In this study, we evaluated the antiproliferative and proapoptotic effects of triphala, a widely used formulation in Indian traditional medicine, on HCT116 colon cancer cells and human colon cancer stem cells (HCCSCs). The total phenolic content, antioxidant activity, and phytochemical composition (LC-MS-MS) of methanol extract of triphala (MET) were also measured. We observed that MET contains a variety of phenolics including naringin, quercetin, homoorientin, and isorhamnetin. MET suppressed proliferation independent of p53 status in HCT116 and in HCCSCs. MET also induced p53-independent apoptosis in HCCSCs as indicated by elevated levels of cleaved PARP. Western blotting data suggested that MET suppressed protein levels of c-Myc and cyclin D1, key proteins involved in proliferation, and induced apoptosis through elevation of Bax/Bcl-2 ratio. Furthermore, MET inhibited HCCSCs colony formation, a measure of CSCs self-renewal ability. Anticancer effects of triphala observed in our study warrant future studies to determine its efficacy* in vivo*.

## 1. **Introduction**

Colon cancer is the second leading cause of cancer deaths in USA. In 2014, an estimated 71,830 men and 65,000 women were diagnosed with colorectal cancer, while 26,270 men and 24,040 women died due to the disease [1]. p53, one of the critical tumor suppressor genes, is mutated in 50−75% of colon cancer cases and marks transition to metastasis [2–4]. 5-Fluorouracil (FU) remains a widely used chemotherapeutic drug in the treatment of colorectal carcinoma; however, its anticancer efficacy is partly attributed to its ability to induce p53-dependent cell growth arrest and apoptosis; consequently, mutations or deletions of p53 can cause cells to become resistant to FU [5]. Similar to other cancers, current viewpoint suggests that colon cancer is a disease of aberrant stem cell populations [6]. Stem cells have the ability to self-renew for many generations, making them live long enough to acquire the mutations necessary to become cancer stem cells (CSCs) [7]. These cells can reproduce themselves and sustain the cancer [8]. The idea that a smaller population of stem cells primarily drives cancer has important implications. For instance, many anticancer therapies are evaluated based on their ability to shrink tumors, but if the therapies are not effective in complete elimination of the CSCs, the tumor will soon grow back [8, 9]. The proliferation and the acquisition of stem cell fates are coordinated by a small number of highly evolutionarily conserved signaling pathways, such as the Wnt signaling. The Wnt pathway proteins are a group of intracellular signaling molecules that constitute the principal driving force behind the biology of the crypt and play an important role in the maintenance and proliferation of stem cell reservoirs [10]. c-Myc and cyclin D1 are oncogenes, the key signatory genes of Wnt signaling, and both function in the stimulation of cell proliferation and in resistance to apoptosis. Coordination of c-Myc with cyclin D1 or its upstream activators may not only accelerate tumor formation but also drive tumor progression to a more aggressive phenotype. Because c-Myc may affect immortalization while cyclin D1 elicits transformation, agents that target c-Myc and cyclin D1 can be good chemopreventive agents [11]. Recently, c-Myc has been recognized as an important regulator of stem cell biology as it may serve as a link connecting malignancy and “stemness” and has central role in cell proliferation, apoptosis, and survival of CSCs [12–14]. CD133-positive colon CSCs were shown to be resistant to the conventional cytotoxic drug FU and the resistance was shown recently to be dependent on Wnt signaling [15].

There is an increasing evidence of a preventive/protective role of dietary plant extracts, especially fruits, vegetables, grains, and herbs, against colon cancer. These foods are rich in bioactive compounds such as polyphenols, carotenoids, and glucosinolates. The chemopreventive properties of these compounds include inhibiting cell proliferation, inducing apoptosis, and scavenging free radicals. Indeed, dietary intervention is emerging as an alternative to prevent the progression of colon cancer mainly due to its potency and reduced toxicity [16, 17]. We have reported previously that bioactive compounds from purple-fleshed potatoes and grapes suppressed cell proliferation and induced apoptosis in prostate cancer cells (LNCaP and PC3 cells) and colon cancer cells (HT29 and HCT116, independent of p53 status) [18–22]. We also reported that resveratrol, a grape bioactive compound, suppressed colon cancer cell proliferation and elevated apoptosis even in the presence of IGF-1 (insulin like growth factor-1, a mitogen elevated during obesity) via suppression of IGF-1R/Akt/Wnt signaling pathways and activation of p53 [19].

Triphala churna is a powdered formulation of bioactive compound rich three myrobalan fruits,* Emblica officinalis *Gaertn. (Amla),* Terminalia chebula* Retz. (Haritaki), and* Terminalia bellirica *Roxb. (Bibhitaki) in equal proportions. This formulation has been extensively used in the traditional Indian system of medicine, Ayurveda, for the treatment of several disorders of the gastrointestinal and cardiovascular systems [23–25]. Triphala has also shown to inhibit the growth of carcinogen induced stomach cancer, thymic lymphoma, and pancreatic cancer in mice [26, 27]. However, there are no studies, to our knowledge, regarding the effects of triphala in the colon and specifically against HCCSCs. The aim of the current study was to characterize the antiproliferative and proapoptotic activities of the triphala bioactives on HCCSCs. We hypothesized that triphala bioactives suppress colon cancer stem cell proliferation and elevate apoptosis via suppressing Wnt signaling pathways and induction of mitochondrial mediated apoptosis.

## 2. **Materials and Methods**

### 2.1. Chemicals

Commercial triphala powder was purchased from Dabur India Ltd. (India). Fetal bovine serum (FBS) was purchased from HyClone (Pittsburgh, PA). BrdU cell proliferation assay kit was obtained from Cell Signaling Technology (Danvers, MA). Gallic acid, Trolox ((±)-6-hydroxy-2,5,7,8-tetramethylchromane-2-carboxylic acid), ABTS (2,2′-azino-bis(3-ethylbenzothiazoline-6-sulfonic acid) diammonium salt), Folin-Ciocalteu reagent, and all other chemicals were obtained from Sigma-Aldrich (St. Louis, MO).

### 2.2. Preparation of Triphala Extracts

Triphala powder was extracted in two ways: (1) serially in a continuous sequence with hexane, acetone, methanol, alcohol, and water or (2) directly with aforementioned solvents, by vortexing and incubating at 4°C overnight in a shaking incubator. After incubation, extracts were collected and centrifuged at 4000 rpm for 10 minutes. Supernatants were collected, filtered through a 0.45 *μ*m PTFE syringe filter (Tisch Scientific, North Bend, OH), and concentrated in a nitrogen evaporator. The concentrated extract was used for quantification of total phenolics and antioxidant ability. Extracts were aliquoted and stored at −80°C until further use.

### 2.3. Total Phenolics in Triphala Extracts

Total phenolic content of the triphala extracts was determined using a modified Folin-Ciocalteu colorimetric method [28, 29]. In a 96-well microplate, 35 *μ*L of extract was combined with 150 *μ*L of 0.2 M Folin-Ciocalteu reagent and was allowed to react for 5 minutes. After adding 115 *μ*L of sodium carbonate solution (7.5% w/v), the mixture was allowed to react further for 30 minutes at 45°C and finally cooled for 1 hour at room temperature. The absorbance was read at 765 nm using a microplate reader (Synergy HT, Biotek) and total phenolics were expressed as milligrams of gallic acid equivalents per gram of sample (mg GAE/g).

### 2.4. Antioxidant Activity of Triphala Extracts

The antioxidant activity was measured using a modified 2,2′-Azino-bis(3-ethylbenzothiazoline-6-sulfonic acid) (ABTS) assay [29–31]. For the ABTS assay, equal volumes of 3 mM ABTS radical and 8 mM potassium persulfate were allowed to react in the dark for at least 16 hours at room temperature to prepare the stock solution. Five mL of the stock solution was mixed with 145 mL of phosphate buffer (pH 7.4) to make the working solution. In a 96-well microplate, 290 *μ*L of the ABTS working solution was mixed with 10 *μ*L of extract and allowed to react for 30 minutes. The absorbance was measured at 734 nm using a microplate reader. The antioxidant activity of the samples was expressed as milligrams of Trolox equivalents per gram of sample (mg TE/g).

### 2.5. LC-MS-MS Analysis

Methanol extract (one *μ*L) of triphala was injected on a Waters Acquity UPLC system. Separation was performed using a Waters Acquity UPLC T3 column (1.8 *μ*M, 1.0 × 100 mm), using a gradient from solvent A (water, 0.1% formic acid) to solvent B (acetonitrile, 0.1% formic acid). Injections were made in 100% A, which was held for 1 minute; then a 12-minute linear gradient to 95% B was applied and was held at 95% B for 3 minutes. The mobile phase was returned to starting conditions over 0.05 minutes and held for 3.95 minutes. Flow rate was kept constant at 200 *μ*L/min for the duration of the run. The column was held at 50°C and samples were held at 5°C. Column eluent was infused into a Waters Xevo G2 Q-TOF MS fitted with an electrospray source. Data was collected in positive ion mode, scanning from 50 to 1200 at a rate of 0.2 seconds per scan, alternating between MS and data dependent acquisition (DDA) MS/MS mode. Collision energy was set to 6 V for MS mode and ramped from 15 to 30 V for MS/MS mode. Calibration was performed prior to sample analysis via infusion of sodium formate solution with mass accuracy within 1 ppm. The capillary voltage was held at 2200 V, the source temperature at 150°C, and the desolvation temperature at 350°C with a nitrogen desolvation gas flow rate of 800 L/hr. Raw data files were converted to mzXML format using massWolf, and the MS/MS spectra were extracted using XCMS and written to msp format using a custom script in the statistical program R [32]. Later msp files were submitted to MassBank database (http://www.massbank.jp/?lang=en) to obtain the data for identification of tentative compounds present in the methanol extract of triphala. These tentative compounds were identified based on high scores according to Sumner et al. [33]. Comparing retention time and mass spectra to analytical standard, we performed metabolite confirmation for naringin.

### 2.6. Cell Lines

Human colon cancer stem cells (HCCSCs, positive for cancer stem cell markers CD133, CD44, CD34, aldehyde dehydrogenase, telomerase, Sox2, cKit, and Lin28) were obtained from Celprogen Inc. (San Pedro, CA). To maintain the cells in their undifferentiated state, HCCSCs maintenance media (10% FBS) and specially coated cell culture flasks (Celprogen) were used. Cells were maintained at 37°C and 5% CO_2_. Cell cultures at approximately 80% confluence were used for all* in vitro *experimental procedures.

Human colon cancer cell lines HCT116 p53^+/+^ and HCT116 p53^−/−^ were a gift from Dr. Bert Vogelstein (School of Medicine, Johns Hopkins University, Baltimore, MD). Cells were maintained at 37°C in a humidified atmosphere with 5% CO_2_ as described earlier [21].

### 2.7. Lentiviral shRNA-Mediated Knockdown of p53 in HCCSCs

HCCSCs were infected with lentiviral particles encoding shRNA targeting p53 (Santa Cruz Biotechnology, Paso Robles, CA) according to the supplier's protocol. Briefly, HCCSCs were infected at a multiplicity of infection of 10 in HCCSC growth medium containing 5 *μ*g/mL of Polybrene at 37°C and 5% CO_2_. After 24 hours, media were replaced with fresh media and the cells were cultured for 2 days. The infected cells were selected in the presence of puromycin (7.5 *μ*g/mL) for 5 days. Protein extract from these cells was run on a gel to confirm complete suppression of p53 as described earlier [34].

### 2.8. Cell Proliferation Assay

The BrdU cell proliferation assay kit was obtained from Cell Signaling Technology (Danvers, MA) and used for the detection of 5-bromo-2′-deoxyuridine (BrdU) incorporated into cellular DNA during cell proliferation. Briefly, 20,000 cells (HCCSC or HCT116) were seeded per well in a 96-well plate; after 24 hours, growth media were removed and cells were treated with triphala extracts in serum-free media at 25, 50, 100, or 200 *μ*g/mL for 20 hours at 37°C. BrdU was then added at a concentration of 10 *μ*M per well, and the plate was incubated at 37°C for an additional 4 hours to allow incorporation of BrdU into cellular DNA (24 hours total). The medium was removed, and the BrdU incorporation was measured according to the manufacturer's protocol. The experiment was performed in triplicate, and the data is expressed as the mean ± S.E.

### 2.9. Colony Formation Assay

Ability of extract to alter the stemness of colon CSCs was evaluated through colony formation assay [35] by counting the number of colonies that can form after treatment. Briefly, 150,000 HCCSCs were seeded per well in a 6-well plate and incubated for 24 hours in complete growth media. After 24 hours, growth media were removed and cells were treated with methanol extract of triphala (MET) in serum-free media at 25, 50, 100, or 200 *μ*g/mL for 24 hours. Cells were collected by trypsinization. After proper dilution of cells, 100 cells were seeded into each well of a new 6-well plate and were incubated for 10 days in complete growth media. At the end of 10 days, media were removed and cells were fixed using a fixing solution (3.7% paraformaldehyde in 70% ethanol) for 10 min. The cells were stained with 0.05% Coomassie blue for 20 minutes and then rinsed with PBS. Stained colonies were counted under dissecting microscope [36].

### 2.10. Western Blot Analysis

HCCSCs were seeded at a density of 2.0 × 10^6^ cells per culture plate in HCCSC growth media for 24 hours. After 24 hours of incubation, the cells were treated with 5-fluorouracil (IC(50) 570 *μ*M or 75 *μ*g/mL) or methanol extract of triphala (MET, IC(50), 104 *μ*g/mL) in HCCSC serum free media and incubated for 24 hours. Cells were used for nuclear and cytoplasmic proteins separation according to the protocol supplied by NE-PER Nuclear and Cytoplasmic Protein Extraction Kit (Thermo Fisher Scientific, Rockford, IL). Cytoplasmic or nuclear protein extracts (40 *μ*g) were incubated at 98°C for 5 minutes and separated by 4–12% Bis-Tris precast gels (Bio-Rad Laboratories, Hercules, CA) at 120 V for 2 hours in MOPS running buffer (Bio-Rad) and electrophoretically transferred to PVDF Immobilon-FL membrane (Millipore, Billerica, MA) at 35 V for 95 minutes in TBS transfer buffer. PVDF membranes were incubated in superblock solution (Thermo Fisher Scientific) for 2 hours at room temperature. Membranes were then incubated with either PARP rabbit monoclonal antibody, Bax rabbit monoclonal antibody (1 : 1000; Cell Signaling Technology), rabbit anti-Bcl-2 antibody, mouse DNA topoisomerase II*β*, mouse polyclonal anti-cyclin D1 antibody, mouse polyclonal anti-c-Myc antibody, or goat polyclonal anti-*β*-actin antibody (1 : 500, Santa Cruz Biotechnology, Paso Robles, CA) for 2 hours at room temperature. Membranes were subsequently washed with TBS with 0.1% Tween 20 and then probed with IR dye conjugated secondary antibodies, donkey anti-goat, goat anti-rabbit, or goat anti-mouse IR dye 800 or IR dye 680 obtained from LICOR Biosciences, Lincoln (1 : 100,000). All antibody dilutions were made in superblock solution. The membranes were scanned using an Odyssey infrared image system (LICOR Biosciences, Lincoln, NE) and the band intensities were quantified using the Odyssey software and normalized to *β*-actin, a loading control for cytosolic proteins, or to DNA topoisomerase II*β*, a loading control for nuclear proteins.

### 2.11. Statistical Analysis

Data are represented as mean ± standard error (SE). Statistical significance was determined by one-way ANOVA, followed by the post hoc Fisher least significant differences test for multiple means comparisons. All statistical analyses were performed using SPSS version 21 (IBM, Armonk, NY). Mean values not sharing the same letter are statistically significant from each other (*p* < 0.05).

## 3. **Results**

### 3.1. Methanol Is the Best Solvent for Extraction of Polyphenols from Triphala Powder with High Antiproliferative Activities

**Figure 1:**
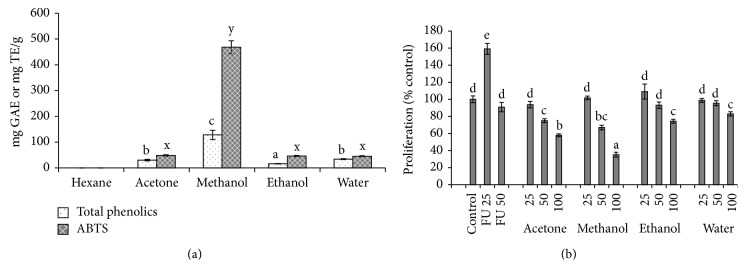
Total phenolics, antioxidant activity, and antiproliferative efficacy of serial solvent extracts of triphala. Total phenolics and antioxidant activity (a) were determined by Folin-Ciocalteu reagent and ABTS assay, respectively, as described in Section 2. Antiproliferative efficacy (b) was determined using BrdU assay by treating the HCCSCs with solvent control: DMSO, FU: 5-fluorouracil (25 and 50 *μ*g/mL), and serial solvent extracts of triphala at 25, 50, or 100 *μ*g/mL for 20 hours. Results were expressed as mean ± SE for three experiments at each time point. GAE: gallic acid equivalents; TE: Trolox equivalents. The error bars with the same letter indicate no significance at *p* < 0.05.

To optimize the extraction of phenolic compounds from triphala powder, we began serial solvent extractions starting with low to high polarity solvents in the order of increasing polarity (hexane, acetone, methanol, ethanol, and water). Highest levels of phenolics were extracted in the methanol solvent compared to other solvent systems as determined by the Folin-Ciocalteu assay. The methanol extract also had the highest antioxidant potential (Figure 1(a)). Antiproliferative activity of triphala extracts was tested against HCCSCs and were compared against the standard cytotoxic drug 5-fluorouracil (FU). FU elevated cell proliferation in HCCSCs compared to control at low concentrations (25 *μ*g/mL) similar to earlier studies (*p* < 0.05) [15]. The acetone and the methanol fraction of the triphala extract exhibited significant cell growth inhibition compared to control and FU. The methanol fraction had the highest antiproliferative activity compared to other solvent extractions (Figure 1(b)).

**Figure 2:**
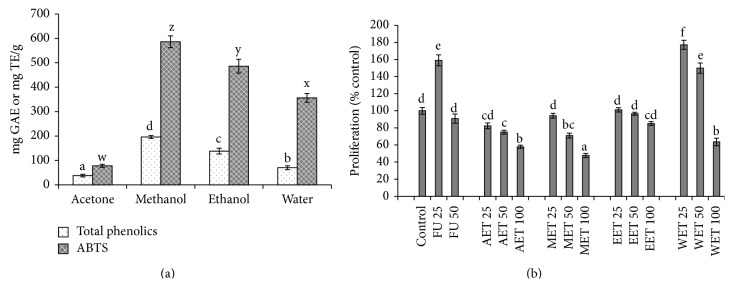
Total phenolics, antioxidant activity, and antiproliferative efficacy of direct solvent extractions of triphala. Total phenolics and antioxidant potential (a) were determined by Folin-Ciocalteu reagent and ABTS assay, respectively. Antiproliferative efficacy (b) was determined using BrdU assay by treating HCCSCs with solvent control: DMSO, FU: 5-fluorouracil (25 or 50 *μ*g/mL), and solvent extracts of triphala at 25, 50, or 100 *μ*g/mL for 20 hours. AET: acetone extract of triphala, MET: methanol extract of triphala, EET: ethanol extract of triphala, and WET: water extract of triphala. Results were expressed as mean ± SE for three experiments at each time point. GAE: gallic acid equivalents; TE: Trolox equivalents. The error bars with the same letter indicate no significance at *p* < 0.05.

To understand whether direct solvent extraction yields higher levels of phenolics compared to serial solvent extraction, the triphala powder was extracted separately with the individual solvents—acetone, methanol, ethanol, and water. Similar to serial extraction, the methanol solvent extracted the highest level of phenolics (Figure 2(a)). Methanol extract also had the highest antioxidant potential and also showed greater antiproliferative activities compared to acetone, ethanol, and water extracts (Figure 2(b)). Thus, the methanol extract of triphala (MET) was used for further studies. Interestingly, although the ethanol and the water extracts had significantly higher total phenolics and antioxidant activity compared to acetone, the antiproliferative effects were not significantly higher.

### 3.2. LC-MS-MS Analysis

Methanol extract of triphala (MET) was used for LC-MS-MS analysis for the identification of the major bioactive compounds. Phenolic acids, flavonoids, alkaloids, and anthocyanins present in the extract are listed in Table 1. The tentative compounds identified in MET were hypaconitine, homoorientin, naringin, embinin, pseudopelletierine, xanthohumol, atomoxetine, acaciin, quercetin, malvidin-3-O-beta-D-galactoside, icariin, nodakenin, geniposide, and so forth (Table 1). Among these bioactives, MET contains naringin, quercetin, homoorientin, isorhamnetin, and hypaconitine in high concentrations. Naringin had the highest relative abundance and we confirmed the presence of naringin in the triphala extract by comparing retention time and mass spectra with pure naringin (data not shown).

### 3.3. Methanol Extract of Triphala Suppressed Proliferation in HCCSCs and Cancer Cells (HCT116) in a p53-Independent Manner

**Figure 3:**
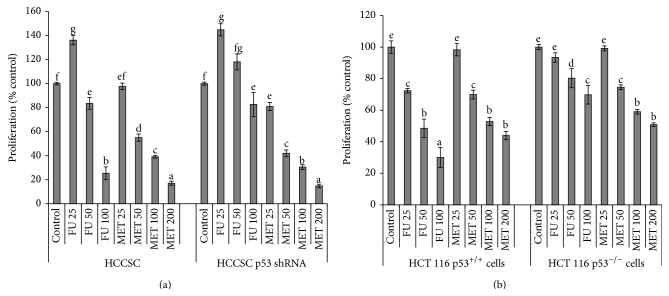
Antiproliferative efficacy of methanol extract of triphala (MET) on HCCSCs with functioning p53 and shRNA-attenuated p53 (a) and p53^+/+^ and p53^−/−^ cells of HCT116 (b). Cells were treated with either 5-fluorouracil (FU, 25, 50, or 100 *μ*g/mL) or MET (25, 50, 100, or 200 *μ*g/mL) for 20 hours and BrdU assay was performed as described in methods. Results were expressed as mean ± SE for three experiments at each time point. The error bars with the same letter indicate no significance at *p* < 0.05.

To investigate dose-dependent antiproliferative activities of MET, we performed BrdU assay on HCCSCs and HCT116 cells treated with increasing concentrations (25, 50, 100, or 200 *μ*g/mL) of MET for 20 hours. FU included in the study served as a positive control. Our results demonstrated that suppression of cell proliferation was elevated with increased concentration of triphala extract on both cell lines in a dose-dependent manner (Figures 3(a) and 3(b)). IC(50) of MET against HCCSCs and HCT116 was 104 ± 5 *μ*g/mL and 153 ± 8 *μ*g/mL, respectively. HCCSCs with shRNA-attenuated p53 and HCT116 p53^−/−^ cells were treated with increasing concentrations of MET (25, 50, 100, or 200 *μ*g/mL). A significant suppression (*p* < 0.05) of proliferation was observed in both of these cell lines even in the absence of p53 (Figures 3(a) and 3(b)). It is important to note that the triphala extract suppressed HCCSCs and HCT116 proliferation in a dose-dependent manner even in the absence of p53. In contrast, the FU treatment showed minimal suppression of cell proliferation in the absence of p53 in both cells.

### 3.4. Methanol Extract of Triphala Induced Apoptosis in a p53 Independent Manner

**Figure 4:**
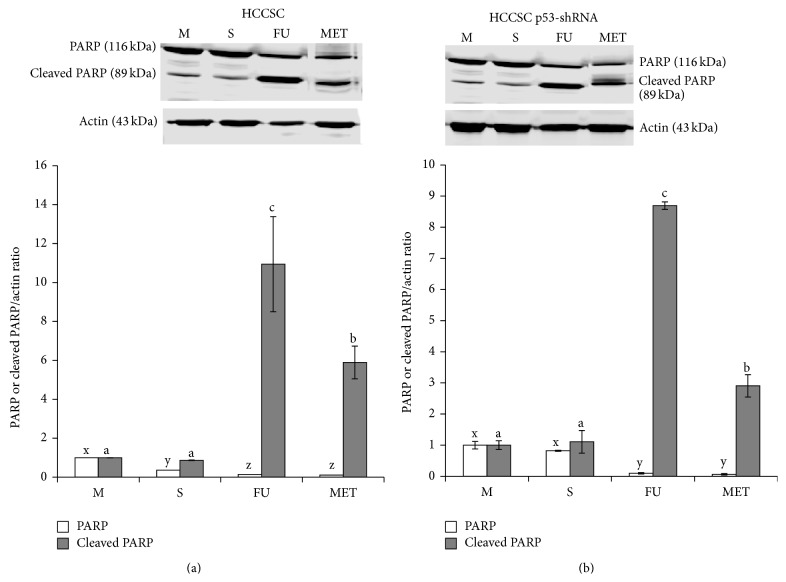
Effect of methanol extract of triphala (MET) on apoptosis of HCCSCs (a) and HCCSC p53-shRNA cells (b). Cells were treated with IC(50) of 5-fluorouracil (75 *μ*g/mL) and MET (104 *μ*g/mL) and apoptosis was analyzed by western blot. Data are representative of three individual experiments; a representative blot is presented. M: media control, S: solvent control, FU: 5-fluorouracil, and MET: methanol extract of triphala. The error bars with the same letter indicate no significance at *p* < 0.05.

The ability of MET to induce apoptosis in HCCSCs and cells with shRNA-attenuated p53 was measured by PARP cleavage, the hallmark of apoptosis, using western blotting.  Figure 4 shows the induction of PARP cleavage as accumulation of cleavage fragment (89 kDa) in MET treated cells. Triphala extract (MET) induced apoptosis in HCCSCs independent of p53 was supported by increased PARP cleavage (Figures 4(a) and 4(b)).

### 3.5. Methanol Extract of Triphala Induces Apoptosis by Elevating Bax/Bcl-2 Ratio

**Figure 5:**
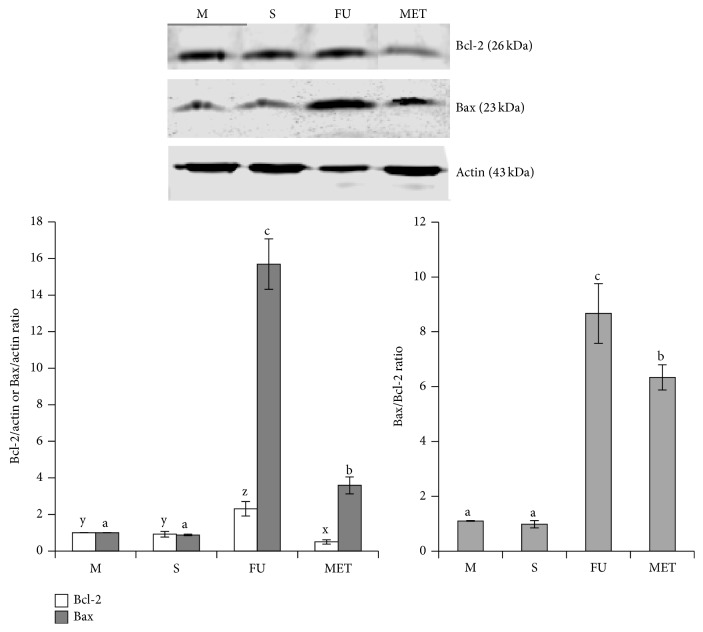
Effect of methanol extract of triphala (MET) on pro- and antiapoptotic protein, Bax, and Bcl-2 expression. HCCSCs were treated with MET (104 *μ*g/mL) and 5-fluorouracil (75 *μ*g/mL). Cytoplasmic proteins were extracted by using NE-PER cytoplasmic extraction kit (Pierce) and then analyzed using western blotting for proapoptotic protein, Bax, and antiapoptotic protein, Bcl-2. Data are representative of three individual experiments; a representative blot is presented. M: media control, S: solvent control, FU: 5-fluorouracil, and MET: methanol extract of triphala. The error bars with the same letter indicate no significance at *p* < 0.05.

To evaluate the mechanism implicated in the triphala-induced apoptosis, we measured intrinsic apoptotic signaling pathway proteins in HCCSC following treatment with 104 *μ*g/mL of MET or 570 *μ*M (75 *μ*g/mL) of 5-fluorouracil. Triphala (MET) treated HCCSCs showed increased Bax and decreased Bcl-2 protein levels in the cytoplasm and increased Bax/Bcl-2 ratio (Figure 5). Although FU treatment elevated Bcl-2 levels, Bax/Bcl-2 ratio was the highest in the FU group.

### 3.6. Methanol Extract of Triphala Suppresses the Stemness of HCCSCs and Suppressed c-Myc and Cyclin D1 Proteins

**Figure 6:**
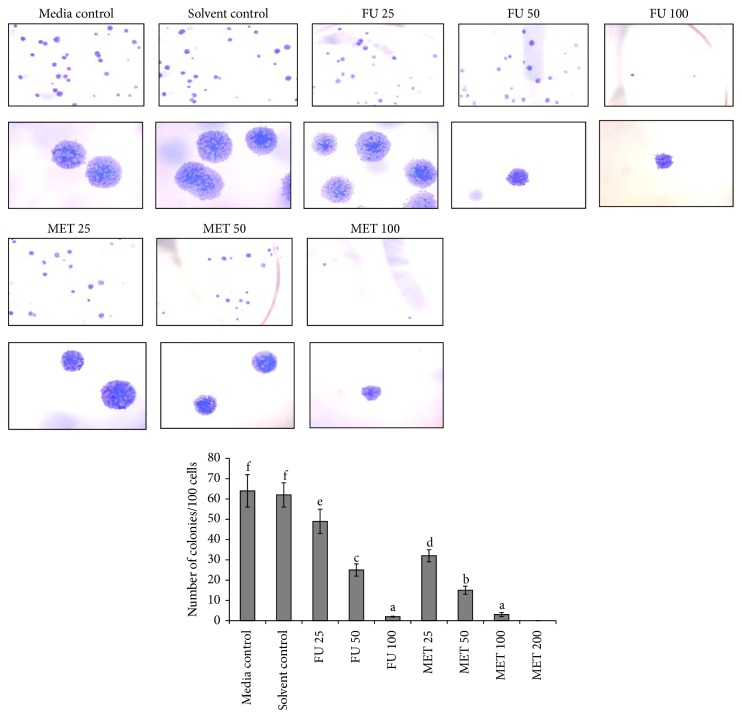
Effect of methanol extract of triphala (MET) on the stemness of HCCSCs. Cells were treated with 5-fluorouracil (FU, 25, 50, or 100 *μ*g/mL) or MET (25, 50, 100, or 200 *μ*g/mL) for 24 hours and colony formation assay was performed. Results were expressed as mean ± SE for three experiments at each time point. The error bars with the same letter indicate no significance at *p* < 0.05.

**Figure 7:**
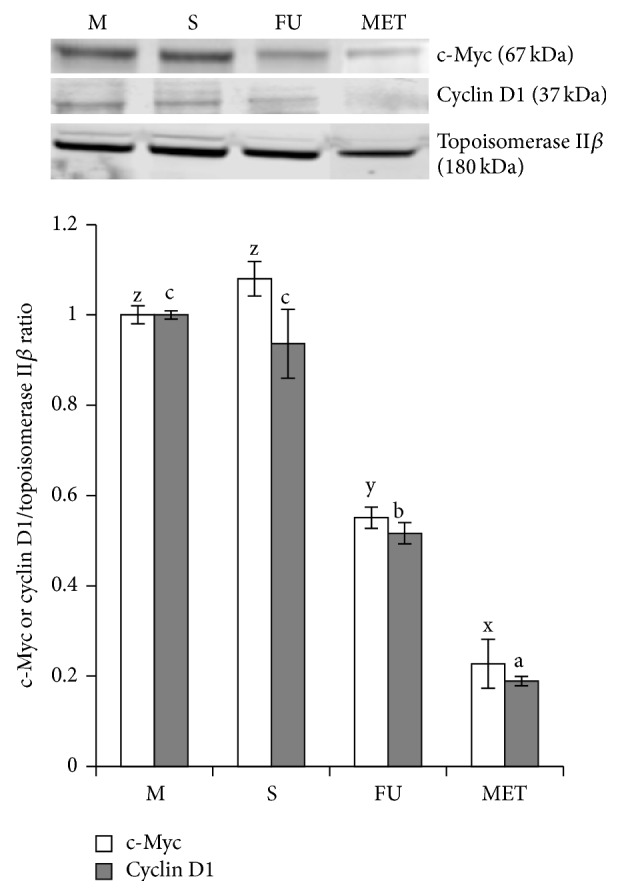
Effect of methanol extract of triphala (MET) on c-Myc/cyclin D1 in HCCSCs. HCCSCs were treated for 24 hours with MET (104 *μ*g/mL) and 5-fluorouracil (FU, 75 *μ*g/mL). Nuclear proteins were extracted by using NE-PER cytoplasmic extraction kit and then analyzed by western blot for c-Myc, cyclin D1, and DNA topoisomerase II*β*. Data are representative of three individual experiments. M: media control, S: solvent control, FU: 5-fluorouracil, and MET: ethanol extract of triphala. The error bars with the same letter indicate no significance at *p* < 0.05.

The stemness (self-renewal) of HCCSCs treated with 25, 50, 100, or 200 *μ*g/mL of MET for 24 hours was evaluated using the colony-forming assay [35]. Our results demonstrated that suppression of colony formation by MET was dose-dependent (Figure 6). MET completely inhibited the formation of colonies at 200 *μ*g/mL. To understand the effect of triphala extract on stemness, we measured expression of c-Myc and cyclin D1, the key target genes of Wnt pathway using western blotting. We observed a significant decrease in the expression of c-Myc and cyclin D in the nucleus (*p* < 0.05) (Figure 7). This may explain how triphala extract suppresses colon cancer stem cell proliferation and colony formation ability.

## 4. **Discussion**

Naturally occurring phytochemicals are widely used in the traditional Indian medicinal system of Ayurveda for treatment of a variety of diseases [37]. These phytochemicals affect a myriad of intracellular targets, and this quality makes them desirable as chemotherapeutic agents against cancer [14, 17, 18, 20, 22, 38–42]. Triphala is an antioxidant-rich herbal formulation containing polyphenols like naringin, quercetin, homoorientin, isorhamnetin, hypaconitine, acaciin, and so forth (Table 1). In the present study, we tested different solvents for extracting these compounds from triphala and identified methanol as the best solvent for extracting the highest levels of bioactive compounds from triphala. Further, we investigated the antiproliferative and proapoptotic effects of methanolic extract of triphala (MET) against HCCSCs and HCT116 cells. Triphala demonstrated dose-dependent antiproliferative properties in HCCSCs and HCT116 colon cancer cells and proapoptotic properties in HCCSCs independent of p53 status of the cells (Figures 3 and 4). We also observed increased expression of p53-responsive Bax (and Bax/Bcl-2 ratio) in HCCSCs (Figure 5). Bax forms a heterodimer with Bcl-2 and functions as an apoptotic activator by increasing the opening of the mitochondrial voltage-dependent anion channel, which leads to the loss in membrane potential and the release of cytochrome c [43–45]. This indicates that triphala induces intrinsic apoptotic signaling pathway [46, 47]. p53 is considered the “the guardian of the genome” and it plays a critical role in tumor suppression by inducing growth arrest, apoptosis, and senescence, as well as by blocking angiogenesis. In addition, p53 generally confers the cancer cell sensitivity to chemoradiation. p53 is thus an attractive target for mechanism-driven anticancer therapies. However, mutational inactivation of p53 is the most frequent event found in 50% of human cancers [48, 49]. Our results showing that triphala exerts its biological efficacy in human colon cancer cells independent of their p53 status are extremely important outcomes for future translational potential in preventive intervention of colon cancer in the clinic; however, preclinical studies in mouse models of colon cancer are required to establish* in vivo* efficacy.

Triphala extract inhibited the stemness (measured using colony formation assay) of colon cancer stem cells in a dose-dependent manner (Figure 6). Wnt signaling is crucially important in maintaining stemness in normal colon stem cells. Wnt signaling activates the TCF/LEF family to promote a progenitor-like gene expression signature (c-Myc and cyclin D1) and is a common pathway that is deregulated in most colon cancers [14, 50]. Treatment with triphala extract (MET) suppressed c-Myc and cyclin D1 expression (Figure 7) and resulted in reduced cell proliferation and colony formation (stemness, Figure 6) [51]. Recent research shows that dietary compounds including grape seed extract, curcumin, lycopene, and resveratrol are promising chemopreventive agents against various types of cancers owing to their direct and indirect effects on cancer stem cell self-renewal pathways [17, 50]. Triphala has a high content of flavonoids like naringin, quercetin, homoorientin, and hypaconitine as shown in Table 1. Specifically, naringin has shown to inhibit growth potential of triple negative breast cancer cells by modulating the Wnt/*β*-catenin pathway [52]. Naringin has also shown to target cyclin D1 in two studies in vascular smooth muscle cells [53, 54]. Flavonoids have shown to block the TCF/LEF family of transcription factors in the nucleus to decrease the expression of oncogenic proteins (c-Myc and cyclin D1) [55, 56]. Triphala extract induced c-Myc and cyclin D1 downregulation might explain decreased HCCSC proliferation and stemness [57]. As triphala is a mixture of these compounds, we believe that the anticancer effect of triphala is due to additive/synergistic effects of the polyphenols. Relatively high doses of single bioactive agents may show potent anticarcinogenic effects; however, the synergistic interactions between different dietary ingredients that potentiate the activities of any single constituent better explain the observed benefits of whole foods and diets in many epidemiological studies [58, 59]. Future studies will employ knockout models to understand role of c-Myc and cyclin D1 in the anticancer efficacy of triphala.

## 5. **Conclusions**

In this study, our findings indicated that triphala was an antioxidant-rich herbal formulation containing polyphenols such as naringin, quercetin, homoorientin, Isorhamnetin, hypaconitine, and acaciin. Triphala (MET) exhibited dose-dependent antiproliferative properties in cancer stem cells (HCCSC) and cancer cells (HCT116) and proapoptotic properties in HCCSCs independent of p53 status of the cells. Furthermore, MET inhibited HCCSCs colony formation (stemness). MET suppressed proliferation/stemness might involve interplay of c-Myc/cyclin D1 signaling. Apoptosis induction by triphala was via the mitochondrial apoptotic signaling characterized by elevated Bax/Bcl-2 ratio. These results indicate that triphala (MET) may serve as an effective chemopreventive agent against colon cancer.

Since triphala is already in human use as a dietary supplement for its several health benefits, findings of our present study on the anticancer effects of triphala, even against HCCSCs, suggest that detailed* in vivo* preclinical studies are warranted to evaluate the mechanism-based anticancer efficacy of triphala against advanced stages of colon cancer. Further, investigation of triphala for its potential use as an adjunct to conventional chemotherapy in the management of colon cancer is also warranted.

## Figures and Tables

**Table 1:** Tentative compounds determined by LC-ESI-QTOF-MS-MS in methanol extract of triphala (MET).

No.	*m*/*z*	RT^a^	Score^b^	Tentative identified molecule^c^	MassBank ID	Molecular formula	Exact mass	Area under curve
1	617.50	15.66	0.853	Hypaconitine	TY000048	C33H45NO10	615.30	24347
2	345.05	0.52	0.805	Naringin^*∗*^	PB000804	C27H32O14	580.18	139768
3	607.21	5.99	0.800	Embinin	TY000131	C29H34O14	606.19	6610
4	135.95	19.94	0.760	Pseudopelletierine	KOX00830	C9H15NO	153.12	560
5	687.14	1.38	0.750	Xanthohumol	CE000112	C21H22O5	354.15	9876
6	186.96	19.49	0.743	Atomoxetine	EA284610	C17H21NO	255.16	363
7	593.27	12.13	0.737	Acaciin	PR020027	C28H32O14	592.18	19729
8	493.25	6.77	0.695	Malvidin-3-O-beta-D-galactoside	PR020065	C23H25O12	493.13	4302
9	449.07	4.59	0.686	Isorhamnetin	TY000220	C16H12O7	316.06	33552
10	471.02	3.01	0.681	Homoorientin	PR040134	C21H20O11	448.10	75557
11	364.02	2.06	0.680	2-{2-Benzimidazol-2-yl-1-[(4-methylphenyl)methyl] ethyl}benzimidazole	BML80240	C24H22N4	366.18	330
12	609.27	11.61	0.674	Reserpine	CE000152	C33H40N2O9	608.27	429
13	97.97	19.71	0.672	Sotalol	EA017004	C12H20N2O3S	272.12	1287
14	130.01	0.08	0.671	Epoxiconazole	EA009507	C17H13ClFN3O	329.07	722
15	677.38	9.35	0.670	Icariin	TY000037	C33H40O15	676.24	2828
16	409.16	7.46	0.670	Nodakenin	TY000089	C20H24O9	408.14	6173
17	303.05	4.63	0.660	Quercetin	PB006206	C15H10O7	302.04	82528
18	557.09	13.91	0.640	Fukugetin	TY000181	C30H20O11	556.10	498
19	411.36	10.65	0.616	Geniposide	TY000059	C17H24O10	388.14	3366

^a^The retention times (RT) (in minutes) are according to the LC-MS system. ^b^Similarity score of retrieved spectrum to a query data from the MassBank database. ^c^Tentative molecules were identified based on high score [33]. ^*∗*^Confirmed using comparison on retention time and mass spectra with analytical standard naringin.

## Abbreviations

PARP: Poly ADP ribose polymerase
BrdU: Bromodeoxyuridine
Bax: Bcl-2 associated X protein
Bcl-2: B cell lymphoma 2 protein
Wnt: Wingless/integrated
MET: Methanol extract of triphala
FU: 5-Fluorouracil.
